# Oral Pathogen *Porphyromonas gingivalis* Can Escape Phagocytosis of Mammalian Macrophages

**DOI:** 10.3390/microorganisms8091432

**Published:** 2020-09-18

**Authors:** Erik R. Werheim, Kevin G. Senior, Carly A. Shaffer, Giancarlo A. Cuadra

**Affiliations:** 1 Department of Biology, Muhlenberg College, 2400 W. Chew Street, Allentown, PA 18104, USA; tuk72888@temple.edu (E.R.W.); kevinsenior@ufl.edu (K.G.S.); cashaffer@muhlenberg.edu (C.A.S.); 2 Lewis Katz School of Medicine, Temple University, 3500 North Broad Street, Philadelphia, PA 19140, USA; 3 Graduate Program in Biomedical Sciences, College of Medicine, University of Florida, P.O. Box 100215, Gainesville, FL 32610, USA

**Keywords:** *P. gingivalis*, THP-1, RAW 264.7, oral, macrophage, phagocytosis, escape, periodontitis, *PG0717*

## Abstract

Macrophages are phagocytic cells that play a key role in host immune response and clearance of microbial pathogens. *Porphyromonas gingivalis* is an oral pathogen associated with the development of periodontitis. Escape from macrophage phagocytosis was tested by infecting THP-1-derived human macrophages and RAW 264.7 mouse macrophages with strains of *P. gingivalis* W83 and 33277 as well as *Streptococcus gordonii* DL1 and *Escherichia coli* OP50 at MOI = 100. CFU counts for all intracellular bacteria were determined. Then, infected macrophages were cultured in media without antibiotics to allow for escape and escaping bacteria were quantified by CFU counting. *P. gingivalis* W83 displayed over 60% of the bacterial escape from the total amount of intracellular CFUs, significantly higher compared to all other bacteria strains. In addition, bacterial escape and re-entry were also tested and *P. gingivalis* W83, once again, showed the highest numbers of CFUs able to exit and re-enter macrophages. Lastly, the function of the *PG0717* gene of *P. gingivalis* W83 was tested on escape but found not related to this activity. Altogether, our results suggest that *P. gingivalis* W83 is able to significantly avoid macrophage phagocytosis. We propose this ability is likely linked to the chronic nature of periodontitis.

## 1. Introduction

Periodontitis is a chronic inflammatory condition caused by bacterial invasion of the oral epithelial tissue around the tooth, leading to tooth loss [1]. A precursor condition to periodontitis is gingivitis, an infection of the gums, where a disruption of the bacterial homeostasis in the tissue surrounding the tooth causes inflammation [2]. This inflammation can increase the chance of oral bacteria entering the bloodstream due to dilation of the oral vasculature [3]. Although the presence of bacteria in the bloodstream may be fleeting, there is the possibility that bacteria may reach a variety of target organs [3].

Macrophages are a variety of cells found in all tissues and exhibit a diverse range of functions such as maintaining homeostasis, supporting development, regulation of tissue modeling and repair, and immune surveillance [4,5,6,7,8]. Macrophages can derive from circulating blood monocytes that stem from bone marrow precursors, establish themselves in tissues, and operate independent of bone marrow originates [4,6,9]. They have functional plasticity and the ability to constantly alter their phenotypes according to environmental stimuli [10,11,12,13]. Either membrane-bound or soluble factors in the microenvironment can drive macrophage differentiation to the classical M1 pro-inflammatory macrophages or to the opposite extreme M2 anti-inflammatory cells [6,12,14,15]. M1 macrophages are commonly polarized by granulocyte-monocyte colony stimulating factor (GM-CSF), interferon (IFN) γ, and lipopolysaccharide (LPS) [14,16,17,18]. M1 macrophages express high levels of pro-inflammatory cytokines including tumor necrosis factor (TNF) α, interleukin (IL) 1β, and IL-6 [19], which cause the inflammation and tissue destruction observed in periodontitis [20,21,22]. Typically, unpolarized M0 macrophages generate cytokines like TNF α as well; however, the levels of production are much lower than M1 macrophages [17,18].

Macrophages’ professional phagocytic abilities also allow them to play a key role in inflammation and host immune response [4,7,23,24]. In the early stages following infection, macrophages use a variety of receptors to recognize microbial agents [25]. An example of these receptors are toll-like receptors (TLR)2 and TLR4, which have been shown to be essential in the detection and response to bacterial products [26]. After recognition, a signaling cascade causes remodeling of the actin cytoskeleton that allows the macrophage to extend its cell membrane around the foreign agent to engulf it [5,24,25]. Upon engulfment, the microbe is found in a structure called the phagosome [27]. In order to destroy the microbe, the phagosome must undergo phagosome maturation, which is a series of fusion and fission interactions between the phagosome, early and late endosomes, and lastly, lysosomes [25,28]. At the end of the process, the mature phagosome, also known as a phagolysosome, has an internal acidic environment and contains several hydrolytic enzymes such as cathepsins, proteases, lysozymes, and lipases aimed to digest microbial structures [25,28,29]. Additionally, phagolysosomes contain scavenger molecules like lactoferrin and NADPH oxidase which interfere with microbial needs and generate reactive oxygen radicals that disrupt microbial biochemistry [25,30]. However, despite the phagolysosomes’ superior ability to capture and degrade microbial agents, there are several pathogens that have developed mechanisms to interfere with their functions [7,25,31,32].

A particular species of bacteria known to circumvent many immune functions in a variety of cell types is *Porphyromonas gingivalis* [33,34,35]. *P. gingivalis* is an anaerobic, Gram-negative, rod-shaped pathogen typically found in the oral cavity [33,36]. It has numerous virulence factors that contribute to its pathogenicity such as cysteine proteinases, hemagglutinins, gingipains, lipopolysaccharide (LPS), and fimbriae [33,37,38,39,40]. *P. gingivalis* is a strong causal agent in the development of periodontitis [33,36,41,42,43,44]. This pathogen can sustain chronic infections by expressing unconventional, heterogenous LPS molecules that can inhibit TLR4 receptors, allowing *P. gingivalis* to evade or inhibit antimicrobial functions associated with the specific TLR [33,45,46]. In addition, *P. gingivalis* can disrupt TLR2 crosstalk interactions with other innate receptors such as the complement 5a receptor (C5aR), C3R, and the CXC-chemokine receptor 4 [33,47,48,49]. The ability of *P. gingivalis* to endure the inflammatory response caused by periodontal infection has been shown to be an important risk factor for numerous other conditions. Inflammation leads to sores and gum bleeding where the bacteria can enter the bloodstream [3]. *P. gingivalis* has been correlated to the acceleration and development of cardiovascular disease, as well as other systemic diseases such as diabetes, arthritis, respiratory infections, and Alzheimer’s [50,51,52,53,54,55,56,57,58,59,60,61,62,63,64,65,66,67,68]. Of importance, several biomarkers including malondialdehyde [69], asymmetric dimethylarginine, and c-reactive proteins [70], have been reported to be elevated among individuals suffering from periodontitis and/or cardiovascular disease, thus serving as potential indicators of complications with these clinical conditions.

*P. gingivalis* novel virulence gene *PG0717*, of the W83 strain, encodes for a hypothetical lipoprotein within the periplasmic space [71,72]. *PG0717* is upregulated during invasion of human coronary artery endothelial cells (HCAEC), hinting that it may be involved in virulence [72]. *P. gingivalis* W83 displays high levels of adherence, invasion, and persistence in HCAEC. However, deletion of the *PG0717* gene does not affect the ability to adhere and invade HCAEC [72,73]. Rather, it impairs the ability of the bacteria to overtake the HCAEC autophagic pathway and induce autophagy in Saos-2 sarcoma cells [72]. Deletion of this gene does not affect capsule or lipid A structure, but expression of arginine and lysine gingipains are reduced [72]. *PG0717* appears to have pleiotropic effects on *P. gingivalis* W83, which affect microbial induced manipulation of host responses that play roles in infection control and clearance [72].

*P. gingivalis* possesses the ability to enter, exit, and re-enter human oral keratinocytes (HOKs) [74]. Additionally, *P. gingivalis* seems to be able to do the same cycle of entry, exit, re-entry, and multiplication in KB epithelial cells, endothelial cells, and smooth muscle cells [75]. Since *P. gingivalis* possesses the ability to enter and exit a variety of cells, perhaps there is a potential ability of this pathogen to escape professional phagocytes such as macrophages. To test this hypothesis, we designed experiments to investigate the ability of *P. gingivalis* to escape M0 and M1 macrophages immediately after engulfment and compare it to the escape of *Escherichia coli* (Gram-negative rod) and *Streptococcus gordonii* (Gram-positive oral species). Such ability may explain the chronic nature of periodontitis and other *P. gingivalis*-related infections outside the oral cavity. In addition, we tested the putative function of the *PG0717* gene in phagocytosis escape. We found a much higher escape index by *P. gingivalis* W83 compared to the other strains tested. *P. gingivalis* W83 escape ability seems to be *PG0717*-independent. Our results demonstrate that *P. gingivalis* has the ability to escape mammalian pro-inflammatory macrophages. These findings are relevant to the chronic aspects of periodontitis since this pathogen is resilient to phagocytosis. 

## 2. Materials and Methods

### 2.1. Culture Methods for THP-1 and RAW Cells

**Figure 1:**
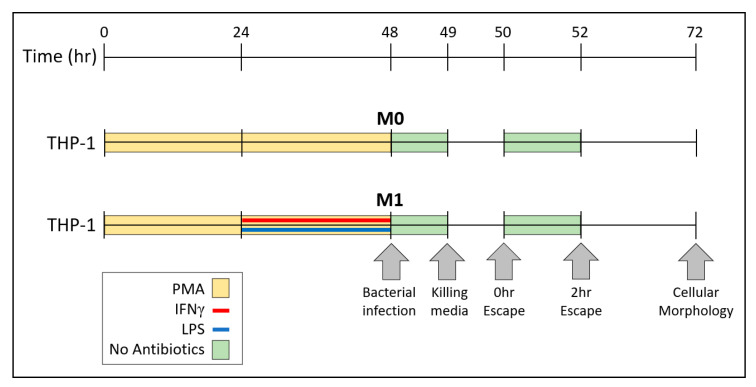
Diagram outlining the protocol of THP-1 differentiation to M0 and M1 macrophages. At 48 h, flow chart of the protocol for bacterial infection, antibiotic protection assay, and bacterial escape in antibiotic-free environments is also indicated. Cellular morphology was determined by microscopy 24 h after bacterial infection, marked at 72 h in the timescale.

THP-1 cells, kindly shared by Dr. Angela Brown from Lehigh University (Bethlehem, PA, USA), were grown and cultured in complete Rosewell Park Memorial Institute (RPMI) 1640 (Gibco, Billings, MT, USA) media containing 10% Fetal Bovine Serum (FBS) and 1:100 penicillin/streptomycin antibiotics at 37 °C 5% CO_2_. Cells were routinely passaged by a 1:5 split with complete RPMI media. For differentiation, THP-1 cells were stimulated to M0 or M1 macrophages following the flow chart in Figure 1. These methods have been previously established [17,18], but with a few alterations. Briefly, two days prior to any experiments, THP-1 cells were counted and seeded at a confluency of 105,000 cells/cm^2^. As shown in Figure 1, cells were differentiated to M0 macrophages by culturing in complete RPMI plus 200 nM phorbol 12-myristate 13-acetate (PMA) at 37 °C 5% CO_2_ for 48 h. To differentiate to M1 macrophages, after the 200 nM PMA for 24 h, cells were then further treated by adding 100 ng/mL lipopolysaccharide (LPS) and 20 ng/mL interferon (IFN) γ. Cultures continued stimulation at 37 °C 5% CO_2_ for the next 24 h (Figure 1).

To assess the cellular morphology of RAW 264.7, THP-1-derived M0, and M1 macrophages, images of cell cultures were obtained at 100× magnification with a Nikon Eclipse TE2000-U inverted microscope equipped with a Nikon Digital Sight DS-Fi1 camera and NIS Elements Imagine Software (Nikon Instruments Inc., Melvin, NY, USA) at the indicated time-points. Cellular morphologies of all three cell types were then compared and confirmed with those from other studies [76,77,78].

RAW 264.7 cells were provided by Dr. John Hoellman from Lincoln Memorial University (Harrogate, TN, USA) and grown and cultured in complete Dulbecco’s Modified Eagle Medium (DMEM) (Gibco, Billings, MT, USA) with 5% FBS and 1:100 penicillin/streptomycin at 37 °C, 5% CO_2_. Cells were routinely passaged at a 1:5 split with complete DMEM. Two days prior to an experiment, RAW cells were counted and seeded at 105,000 cells/cm^2^ and kept in culture at 37 °C, 5% CO_2_ for 48 h.

### 2.2. Culture Methods for Bacteria

Five bacterial strains were used in this study: *E. coli* OP50 (from Dr. Bruce Wightman at Muhlenberg College, Allentown, PA, USA), *S. gordonii* DL1 (from Dr. Robert Burne at the University of Florida, Gainesville, FL, USA), *P. gingivalis* 33277 (from Dr. Richard Lamont at the University of Louisville, Louisville, KY, USA), *P. gingivalis* W83, and isogenic mutant W83Δ0717 (from Dr. Ann Progulske-Fox at the University of Florida, Gainesville, FL, USA). Dr. Progulske-Fox’s team deleted the *PG0717* gene via allelic replacement with plasmid PR-UF1 and other protocols established in the literature [72,79]. All *P. gingivalis* strains were streaked and grown at 37 °C anaerobically in a BACTRON Anaerobic Chamber (Sheldon Manufacturing, Inc. Cornelius, OR, USA) on tryptic soy agar + 5% sheep’s blood + 1 μg/mL menadione (vitamin K). Additionally, *P. gingivalis* W83 was selected with 20 μg/mL gentamicin and *P. gingivalis* W83Δ0717 was selected with 20 μg/mL gentamicin + 10 μg/mL erythromycin. *E. coli* OP50 and *S. gordonii* DL1 were streaked and grown at 37 °C, 5% CO_2_ on tryptic soy agar. Prior to experiments, bacterial colonies from corresponding agar plates were transferred to broths as follows. *P. gingivalis* strains were grown overnight in 4 mL of tryptic soy broth + 1 mg/mL yeast extract + 5 μg/mL hemin + 1 μg/mL menadione (TSBY) anaerobically at 37 °C using the same selection methods as above. *S. gordonii* and *E. coli* were also grown in TSBY at 37 °C 5% CO_2_ overnight without any antibiotic selection. Hours before an experiment, *S. gordonii* and *E. coli* were inoculated from corresponding overnight cultures into fresh cultures with an inoculum size of 1% (40 µL) in 4 mL of TSBY. *S. gordonii* and *E. coli* were grown to optical density (OD) = 1.0 in absorbance units. At this point, *P. gingivalis* overnight cultures were also adjusted to OD = 1.0 in absorbance units. 

Table 1 depicts the genetic differences between *P. gingivalis* strains W83 and 33277. Both strains possess major fimbriae, albeit with different genotypes, affecting their ability to invade cells. *P. gingivalis* 33277 possesses minor fimbriae unlike the W83 strain. However, the latter strain does contain the *PG0717* gene which has been shown to be associated with survival within endothelial cells [72]. *P. gingivalis* W83 has been detailed with each gingipain-associated genotype related to high invasive capabilities (Table 1). *P. gingivalis* 33277 gingipains remain to be explored. Each strain holds a label relating to its virulence: W83 is classified as virulent and 33277 is classified as avirulent (Table 1).

### 2.3. Measurements of Bacterial Internalization by CFU Counts

Bacteria at OD = 1.0 were diluted 1:50 in 1 mL of either DMEM or RPMI media containing FBS but no antibiotics. Two-day old cultures of THP-1 M0 or M1 macrophages or RAW 264.7 mouse macrophages were washed 3× with 1 mL of PBS and infected with the above diluted bacteria. Based on CFU counts, these concentrations of bacteria and macrophages lead to a multiplicity of infection (MOI) of 100. Following the flow chart in Figure 1, co-cultures were incubated at 37 °C 5% CO_2_ for 1 h. At the end of the hour, the non-antibiotic media and excess bacteria were removed from all wells and macrophages were washed twice with 1 mL of PBS. After the washes, 1 mL of either DMEM or RPMI media + FBS + 300 μg/mL gentamicin + 200 μg/mL metronidazole + 1:20 penicillin/streptomycin (killing media) were added to all cultures, corresponding to the cell type. Any extracellular bacteria remaining in the wells were killed for 1 h with killing media at 37 °C, 5% CO_2_. By hour 50, according to the flow chart in Figure 1, samples of supernatant were plated on agar to confirm that no extracellular bacteria remained alive (data not shown). RAW 264.7 macrophages and THP-1-derived M0 and M1 macrophages were washed twice with 1 mL of PBS. Macrophages were then lysed using 1 mL of sterile water. During lysing, wells were scraped with sterile pipette tips to help remove cell debris from the surface and liquid was pipetted up and down to mix thoroughly. Lysates were serially diluted and plated onto respective agar plates in triplicates. *P. gingivalis* CFUs were grown to visible colonies anaerobically, as described above. *E. coli* and *S. gordonii* were grown to visible colonies at 37 °C, 5% CO_2_, as described above. CFUs for all strains were counted and values were calculated to total CFUs/well.

### 2.4. Measurements of Bacterial Escape by CFU Counts

Mammalian macrophages and bacteria were cultured and stimulated separately, as described above. Mammalian macrophages were infected with bacteria (MOI = 100) separately as above for 1 h. Killing media were added to kill any extracellular bacteria for 1 h, as described above. After the hour of killing, media were removed, and macrophages were washed twice with 1 mL of PBS. According to the flow chart in Figure 1, by hour 50, half of a milliliter of fresh antibiotic-free media were added per well, and immediately removed into sterile centrifuge tubes. Macrophages were further washed with 0.5 mL of sterile PBS and this wash volume was immediately added to the 0.5 mL supernatant just collected, yielding a total volume of 1 mL (0.5 supernatant + 0.5 PBS). From this tube, serial dilutions were made and plated in triplicates on corresponding agar. To allow for any further escape, a new volume of 0.5 mL antibiotic-free media was added to macrophages and incubated for 2 h at 37 °C 5% CO_2_. By hour 52, according to the flow chart in Figure 1, supernatants were collected, and another 0.5 mL of PBS was used to wash the wells. Both volumes, PBS, and supernatant were mixed again. To count CFUs, samples were diluted and plated as the 50-h samples. Total escaping CFUs were normalized by calculating the percent escape from total intracellular CFUs: $\frac{\mathrm{Supernatant}\;\mathrm{CFU}}{\mathrm{Intracellular}\;\mathrm{CFU}}$ × 100%. 

### 2.5. Assessment of Cellular Morphology

RAW 264.7, THP-1-derived M0, and M1 cells were observed under the light microscope prior to infection and 24 h post-infection. In addition, uninfected but age-matched cultures of all three cell types were used to compare cellular morphology after bacterial challenges. Micrographs of cellular monolayers were acquired with a Nikon Digital Sight DS-Fi1 camera mounted on a Nikon Eclipse TE2000-U inverted microscope and rendered with NIS Elements Imagine Software (Nikon Instruments Inc., Melvin, NY, USA) at 100× magnification.

### 2.6. Measurements of Bacterial Escape and Re-Entry into Macrophages

THP-1 cells were seeded in 24-well plates as well as on 13 mm round tissue-culture-treated coverslips and stimulated to M1 macrophages, as described above (Figure 1). *S. gordonii* DL1, *E. coli* OP50, and *P. gingivalis* W83 and 33277 were grown and adjusted to OD = 1.0, as described above. M1 macrophages in coverslips were infected with bacteria at MOI = 100 for 1 h as above. Excess bacteria were removed by PBS washes as above and killing media were added for 1 h as above. Then, infected M1 macrophages in coverslips were washed 3× with PBS and coverslips were removed from their respective wells using tweezers sterilized with ethanol. Coverslips with infected M1 macrophages were placed upside down into the wells with uninfected M1 macrophages so that infected and uninfected cells were in contact with each other. Macrophages were then incubated for 2 h in antibiotic-free media to allow for escape from macrophages in coverslips and re-entry into macrophages in wells. Coverslips were then removed and discarded. Cells in wells were washed with PBS as before and killing media were added to wells for 1 h. Finally, M1 macrophages in wells were washed with PBS and lysed with sterile water and scraped as above. The lysates were then diluted, plated, and grown as above for CFU counts.

### 2.7. Genotyping of P. gingivalis W83Δ0717 Mutant Strain

In order to confirm the mutation of the *P. gingivalis* W83Δ0717 isogenic mutant strain, we first used the BLAST tool to analyze the primers used in the Reyes et al. study [72]. BLAST analysis demonstrated that these primers align within an open reading frame of *P. gingivalis* W83 (GenBank ID: AE015924.1) [80] that is expressed in reverse, as described by Reyes at al. Then, PCR and gel electrophoresis were performed as follows. *P. gingivalis* W83, 33277 and W83Δ0717 were grown as described above. Colonies of each strain were placed into centrifuge tubes containing 400 µL of nanopure water and boiled in a 100 °C water bath for 10 min to lyse bacteria and release DNA. Samples were centrifuged at 16,000× *g* for 10 min to pellet bacterial debris. Supernatants, containing DNA, were stored at 4 °C. PCRs were conducted using DNA samples, Taq polymerase, nanopure water, and 10 µM primers previously used for manipulations of the *PG0717* gene by Reyes et al. [72] with forward primer 5′-AAAGGGAGACCAGGAAGTCGACTTGTTCTA-3′ and reverse primer 5′-TTGTTTTCGTATGCATCATCATCGTAGTCA-3′. PCR conditions were as follows: denaturation at 95 °C for 30 s, annealing at 62 °C for 30 s, and extension at 72 °C for 60 s in a total 35 cycle. PCR products were separated in a 2% agarose gel containing ethidium bromide and visualized by UV light. PCR product is expected to be 331 base pairs.

### 2.8. P. gingivalis W83 and W83Δ0717 Escape from THP-1-Derived M1 Macrophages

In order to test the importance of the *PG0717* gene in the escape process, M1 macrophages were chosen based on their fully differentiated phenotype for phagocytosis following the same protocol as described above. Briefly, THP-1 cells were seeded in 24-well plates and differentiated to M1 macrophages as described above (Figure 1). *P. gingivalis* W83 and W83Δ0717 were grown anaerobically and adjusted to OD = 1.0 as described above. M1 macrophages were infected with bacteria at MOI = 100 for 1 h as above. Excess bacteria were removed by PBS washes as above and 1 mL of killing media were added for 1 h as above. Then, infected M1 macrophages washed 3× with 1 mL PBS. Antibiotic-free RMPI was added to all wells and immediately removed to quantify escape following the protocol in Figure 1. Macrophages were then incubated for 2 h with antibiotic-free media to allow for further escape. Any escaping bacteria were serially diluted and plated on blood agar as described above to quantify total CFUs.

### 2.9. Statistical Analysis

All experiments were repeated at least twice with *n* = 3 or higher for each independent trial. From each biological replica, CFU plating was done in triplicates or quadruplicates on agar. All quantitative data presented are the average and the SD from one (*n* = 3) or all independent trials (*n* = 6 or higher). Student’s *t*-tests and ANOVAs were performed to obtain *p* values. All *p* values < 0.05 were considered significant.

## 3. Results

### 3.1. Bacterial Growth in TSBY

**Figure 2:**
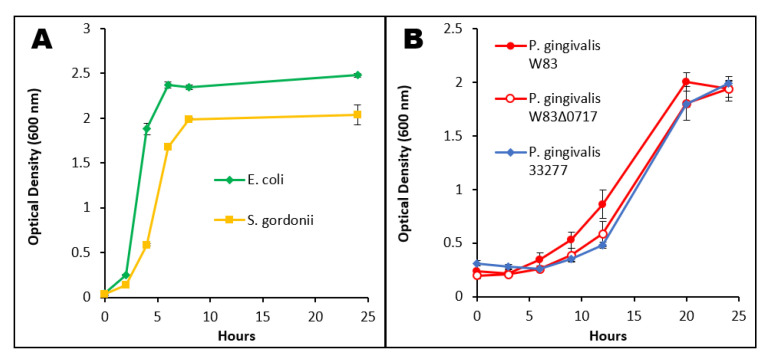
Bacterial growth curves in TSBY, measured by optical density (OD_600 nm_) over the course of 24 h. Commensal species *E. coli* and *S. gordonii* (**A**) were grown aerobically, while *P. gingivalis* strains W83, W83Δ0717, and 33277 (**B**) were grown anaerobically. For each strain, *n* = 3.

To keep nutritional properties consistent between bacteria, all strains were grown in TSBY with menadione. *E. coli* and *S. gordonii* exhibited a lag phase of about 2 h before entering exponential phase (Figure 2A). Both commensal bacteria reached late-exponential phase between 6 and 8 h of growth (Figure 2A). In TSBY, *E. coli* presented slightly faster and higher growth than *S. gordonii* (Figure 2A). All the *P. gingivalis* strains exhibited similar growth patterns (Figure 2B) with respect to each other. The three strains started exponential phase after 8 h (Figure 2B). *P. gingivalis* W83 displayed a slightly faster growth during exponential phase. All strains reached an OD of around 2.0 (units of absorbance) by late-exponential phase, 20 h after inoculation (Figure 2B). Escape testing was always conducted when cells reached late exponential phase and at this time-point, there are no significant differences in the growth and OD of all three *P. gingivalis* strains (Figure 2B).

### 3.2. Cellular Morphology of Mammalian Macrophages

**Figure 3:**
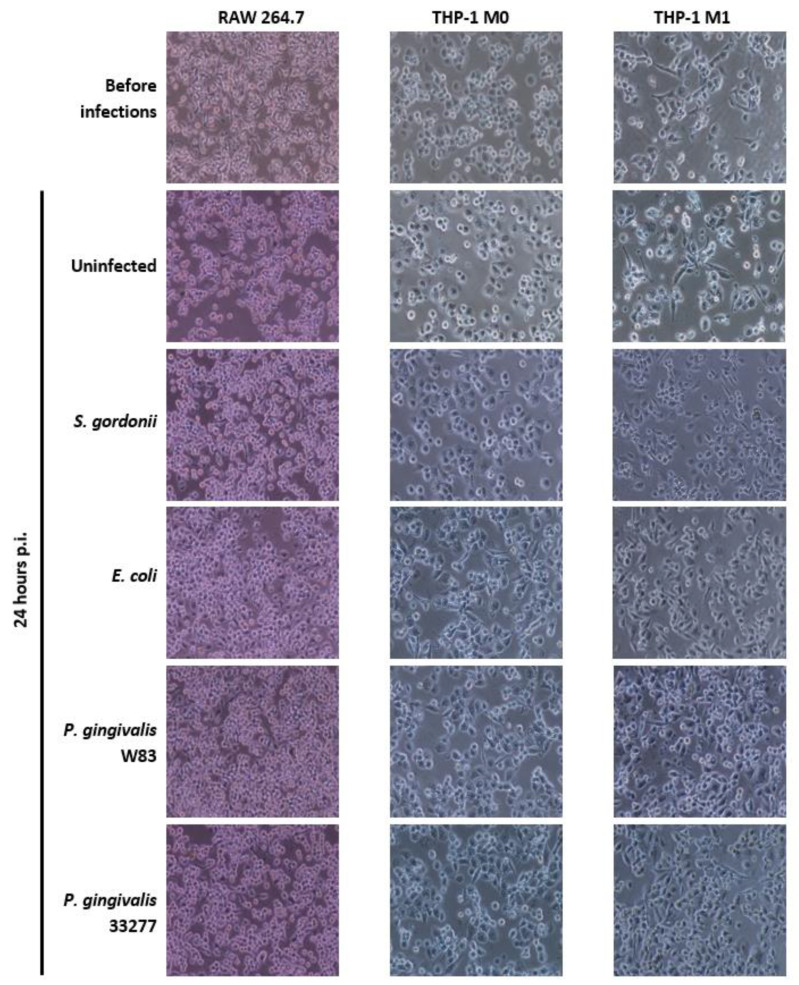
Cellular Morphology: Cellular images of RAW 264.7, THP-1-derived M0, and M1 cells were taken before bacterial infections and 24 h p.i. Representative images of three independent experiments at 100× magnification.

Figure 3 shows images of all macrophage cell types before infections and 24 h post-infection (p.i.) as well as age-matched uninfected cells for comparison. All cell types remained adhered to the surface of the wells, presenting comparable cell numbers, before and after infections. Both RAW 264.7 cells and THP-1-derived M1 cells show extended pseudopodia prior to bacterial challenge (Figure 3). THP-1-derived M0 cells seem more circular before bacterial challenge. All three cell lines present morphologies similar to those published in other studies [76,77,78] confirming that the differentiation to correct cellular phenotypes were achieved. Both RAW 264.7 and M1 cells retain their morphologies before and after infections. On the other hand, M0 macrophages begin to differentiate their morphology to resemble M1 macrophages, especially when exposed to Gram-negative *E. coli* and *P. gingivalis* strains, when compared to age-matched unchallenged M0 cells (Figure 3). Our data indicate that all three cell types are alive and responding to bacterial challenges.

### 3.3. Intracellular Bacteria

**Figure 4:**
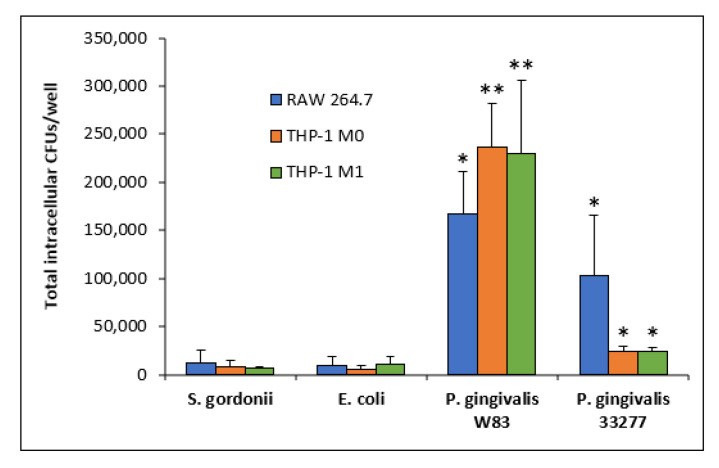
Amounts of intracellular *S. gordonii, E. coli, P. gingivalis* W83, and *P. gingivalis* 33277 CFUs in RAW 264.7 (blue), THP-1 M0 (red), and THP-1 M1 (green) cell cultures. Each value is the average and SD from at least *n* = 6. *, statistically significant compared to *S. gordonii* and *E. coli* (*p* < 0.05). **, statistically significant compared to *S. gordonii*, *E. coli* and *P. gingivalis* 33277 (*p* < 0.05).

Intracellular bacteria were calculated by total CFUs per well. Commensal strains *E. coli* and *S. gordonii* presented less intracellular CFU counts, as high as 11,500 CFUs/well, across all three macrophage types: RAW 264.7, THP-1 M0, and THP-1 M1 (Figure 4). *P. gingivalis* 33277 showed similar intracellular CFU counts, around 23,000/well, in both THP-1 M0 and M1 cell lines to a significantly higher value of 103,000 CFU/well in RAW 264.7 cells (*p* < 0.05) (Figure 4). *P. gingivalis* W83 showed 167,000 intracellular CFUs/well in RAW 264.7 cells, which is significantly higher compared to those of *S. gordonii* and *E. coli* (*p* < 0.05) (Figure 4). Furthermore, *P. gingivalis* W83 intracellular CFU counts reached 236,000/well in THP-1 M0 cells and 230,000/well in THP-1 M1 cells, which are significantly higher than those of all other strains (*p* < 0.05) (Figure 4, Table 2). The total number of macrophages of each cell type is roughly 200,000 cells/well. Intracellular CFUs for strain W83 are nearly at a 1:1 ratio with macrophages in 1 h of interaction. The data suggest that W83 is either engulfed by the macrophages at a faster rate or the pathogen itself invades these immune cells better than all other strains tested.

### 3.4. Escape Assay

**Figure 5:**
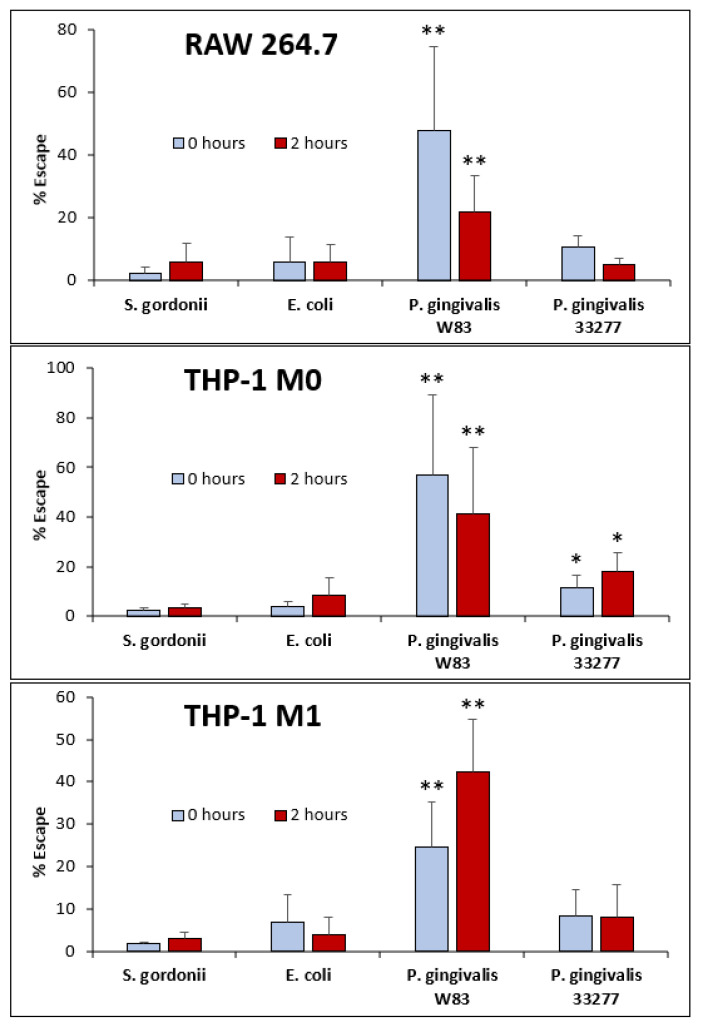
Bacterial Escape % shown at 0 and 2 h across three cell lines: RAW 264.7, THP-1 M0, and THP-1 M1 for bacterial strains *S. gordonii*, *E. coli*, and *P. gingivalis* W83 and 33277. Each value is the mean and SD from at least *n* = 6. *, statistically significant compared to *S. gordonii* and *E. coli* (*p* < 0.05); **, statistically significant compared to *S. gordonii*, *E. coli*, and *P. gingivalis* 33277 (*p* < 0.05).

Bacterial escape was calculated as a percentage of total intracellular bacteria. From RAW 264.7 cells, escape ranged from 2.2% for *S. gordonii* at to 48.1% for *P. gingivalis* W83 at 0 h. At 2 h, *S. gordonii* once again displayed the lowest escape rate at 5.7% and *P. gingivalis* W83 had again the highest escape rate at 21.8% (Figure 5). Similarly, *P. gingivalis* W83 displays significantly higher escape rates from THP-1 M0 and M1 cells compared to the other strains tested (*p* < 0.05) (Figure 5).

In THP-1-derived M0 cells, the escape rate of *P. gingivalis* W83 is 56.9% and 41.0% at 0 and 2 h, respectively; and is significantly higher than those of all other strains (*p* < 0.05). Escape rates for *S. gordonii*, *E. coli*, and *P. gingivalis* 33277 from THP-1 M0 cells were as low as 2.2% and as high as 17.8%. Interestingly, *P. gingivalis* 33277 displayed significantly higher escape rates from THP-1 M0 cells compared to *S. gordonii* and *E. coli* (*p* < 0.05). This difference is not observed in RAW 264.7 nor THP-1 M1 macrophages.

Furthermore, in the case of THP-1-derived M1 cells, the escape rate of *P. gingivalis* W83 is 24.7% and 42.4% at 0 and 2 h, respectively, which is significantly higher than those of all other strains (*p* < 0.05). In M1 cells, the escape ranges for *S. gordonii*, *E. coli*, and *P. gingivalis* 33277 were as low as 1.7% and as high as 8.4%.

### 3.5. Bacterial Escape and Re-Entry in M1 Cells

**Figure 6:**
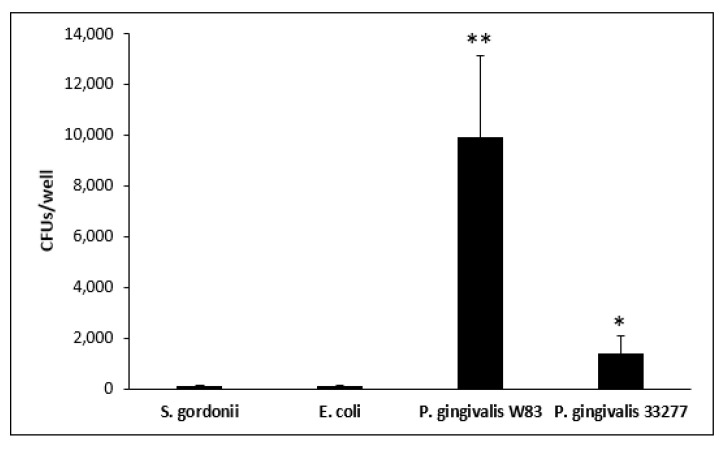
Escape from infected THP-1 M1 cells and re-entry into uninfected THP-1 M1 cells measured by CFU counts per well. Each value is the mean and SD of CFUs found in the second batch of M1 cells from at least *n* = 6. *, statistically significant compared to *S. gordonii* and *E. coli* (*p* < 0.05); **, statistically significant compared to *S. gordonii*, *E. coli*, and *P. gingivalis* 33277 (*p* < 0.05).

Re-entry was tested by allowing escaping bacteria to enter uninfected THP-1-derived M1 cells. *P. gingivalis* W83 exhibited a re-entry of around 10,000 CFUs/well (Figure 6) on average, which is significantly higher than all other strains tested (*p* < 0.01). This number of CFUs represents roughly 6% of the total escaping bacteria (Figure 5, Table 2). *P. gingivalis* 33277 exhibited lower amounts of exit and re-entry with an average CFU count of 1358/well (Figure 6), significantly higher than *S. gordonii* and *E. coli* (*p* < 0.01). These two commensals escaped and re-entered at extremely low values of 83 CFUs for *S. gordonii* and 94 CFUs for *E. coli*. The data indicate that a substantial amount of M1 macrophage-engulfed *P. gingivalis* W83 and 33277 can clearly escape such phagocytic cells and re-enter uninfected ones.

### 3.6. Confirmation of PG0717 Mutation

**Figure 7:**
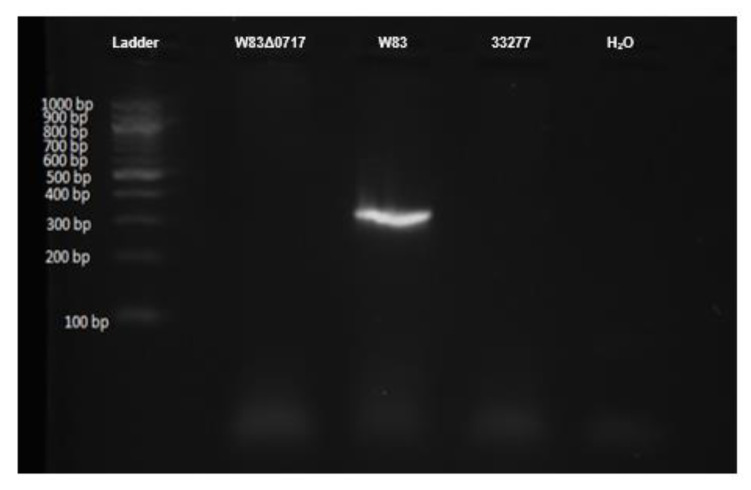
Image of gel electrophoresis after PCR for the *PG0717* gene in *P. gingivalis* W83, 33,227, and W83 W83Δ0717 mutant. H_2_O represents “no template” control. PCR product is expected to be 331 base pairs.

*P. gingivalis* W83, 33,227, and W83Δ0717 mutant were subjected to PCR for the *PG0717* gene followed by electrophoresis. In Figure 7, *P. gingivalis* W83 shows a band at roughly 331 bp according to the primer-amplicon design. In contrast, the 33277 and W83Δ0717 strains do not possess such bands, confirming that the W83Δ0717 strain has the correct mutation for this gene. Based on these results, the remaining experiments were conducted, testing the function of the *PG0717* gene in *P. gingivalis* W83 escape from macrophages.

### 3.7. PG0717 Gene in Bacterial Escape

**Figure 8:**
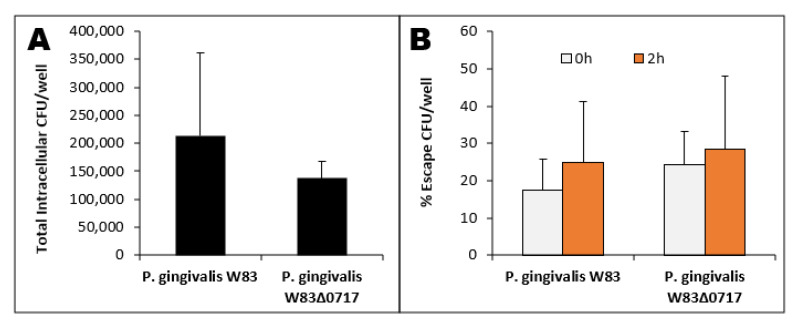
*P. gingivalis* W83 and W83Δ0717 intracellular CFU counts (**A**) and % escape (**B**) from THP-1-derived M1 macrophages.

*P. gingivalis* W83 and W83Δ0717 were tested on growth kinetics (Figure 2B) and showed no significant difference, indicating that this mutation does not affect the growth pattern of the isogenic mutant strain. The *PG0717* gene has been shown to alter the trafficking of intracellular *P. gingivalis* W83 in human coronary artery endothelial cells [72]. Therefore, here, we tested the involvement of the *PG0717* gene in *P. gingivalis* W83 escape from THP-1-derived M1 cells. Both strains were found intracellularly at comparable rates (Figure 8A). In addition, there is no significant difference between the two strains in their ability to escape M1 macrophages (Figure 8B). Our results indicate that the *PG0717* gene is not involved in *P. gingivalis* W83 escape ability from THP-1-derived M1 macrophages.

## 4. Discussion

RAW 264.7 and THP-1-derived M0 and M1 macrophages were infected with different strains of bacteria for 1 h. Commensal strains *E. coli* and *S. gordonii* presented lower intracellular CFU counts across all three macrophage types (Figure 4, Table 2). *P. gingivalis* 33277 presented higher intracellular CFU counts compared to *E. coli* and *S. gordonii.* Although still capable of escape, only as much as 10% of commensal *E. coli* and *S. gordonii* CFUs escaped from all three macrophages. *P. gingivalis* 33277 was able to escape from M1 macrophages at a significantly higher level than commensal strains. Strikingly, *P. gingivalis* W83 exhibited significantly higher intracellular CFUs in almost all macrophages compared to all other strains tested (Figure 5, Table 2). Additionally, *P. gingivalis* W83 also showed significantly higher escape percentages from all macrophages at 0 and 2 h compared to all other strains tested (Figure 5). Moreover, *P. gingivalis* W83 demonstrated the ability to escape THP-1 M1 macrophages and re-enter uninfected ones at a much higher rate than all other strains tested, followed by *P. gingivalis* 33277 (Figure 6, Table 2). In an attempt to discern a mechanism for escape, we analyzed the role of the *PG0717* gene. Although this gene is involved in intracellular trafficking [72], *PG0717* did not seem to be involved in *P. gingivalis* W83 escape from macrophages as the W83Δ0717 isogenic mutant (Figure 7) showed no significant differences in escape ability compared to its parental strain (Figure 8). The results of this study demonstrate that, compared to all bacterial strains tested, *P. gingivalis* W83 displays the highest escape index from the three mammalian macrophage models; *P. gingivalis* W83Δ0717 shows the same level of escape as its parental strain.

Compared to the other strains tested, *P. gingivalis* W83 appears to invade macrophages, rather than being phagocytized (Figure 4). In addition, this microbe escapes the phagocytic cell within 2 h at a high rate (Figure 5) and infects a second macrophage soon after (Figure 6), thus setting this strain apart from the others. The 33277 strain of *P. gingivalis* seems to be able to do this as well, better than commensal bacteria, but at a much lower rate than W83. The genotypic and phenotypic differences between strains W83 and 33277 (Table 1) could account for the observed results in this study. Further studies are required to test this claim.

The escape ability of *P. gingivalis* W83 may have implications in macrophage response to a bacterial infection. RAW 264.7 cells behave similarly to classically activated M1 macrophages when exposed to Gram-negative bacteria and LPS [81]. Both RAW 264.7 and M1 macrophages secrete pro-inflammatory cytokines like IL-1β, IL-6, and TNF-α in response to LPS, unlike M2 macrophages that secrete anti-inflammatory cytokines such as IL-10 and TGF-β, or inactivated M0 macrophages [12,14,81,82,83,84]. Additionally, both RAW 264.7 and THP-1 M1 exhibit a similar elongated morphology in culture, while the M0s appear circular (Figure 3). However, at 24 h p.i., the appearance of the M0 cells exposed to Gram-negative *E. coli* and *P. gingivalis* indicates a change in morphology closer to that of M1s (Figure 3). This could be due to the introduction of LPS, its subsequent binding to TLR4, and the resultant signaling cascade that induces the release of cytokines such as IFN-γ [12,82,83,84]. The combination of IFN-γ and LPS are typical in classical activation of M1 macrophages (Figure 1) and could be, at least in part, the reason why the M0 change in morphology occurred [12,14,82,83]. Moreover, this change in morphology indicates that the macrophages are still alive 24 h p.i. The escape ability of *P. gingivalis* W83 (Figure 5, Table 2) coupled with the activation of M1 macrophages (Figure 3) may play a role in the resultant chronic inflammatory conditions of periodontitis.

*P. gingivalis* is known to cause chronic inflammation that damages the gingival connective tissue, cementum, and alveolar bone [67,68] making use of several virulence factors described in Table 1. These virulence factor together with our results, showing significantly higher escape ratios of *P. gingivalis* W83 (Figure 5, Table 2), may help explain, at least in part, important mechanisms for the chronic aspects of the periodontitis. As the microbe is not well contained by macrophages, the inflammatory response continues, resulting in tissue damage and lesions of the gingiva, where *P. gingivalis* may flourish. Our results imply that even a small infection of *P. gingivalis* can lead to increasing levels of inflammation as one *P. gingivalis* bacterium can infect and exit multiple macrophages (Figure 6, Table 2). These characteristics of *P. gingivalis* may result in sustained and exacerbated inflammation of periodontal tissue in the gingival pocket, leading to signs and symptoms such as bleeding on probing and continuous destruction of oral tissue and bone.

There are numerous other pathogens that have exhibited the ability to escape from the host immune response [80]. *Rickettsia* escapes early from the phagosome through secretion of phospholipase A, which may dissolve the phagosome membrane upon entry into host cells [80]. Another bacteria, *Listeria monocytogenes* utilizes listeriolysin O and two forms of phospholipase C in escape through early lysis of the phagosome [85]. *Shigella* is also able to lyse the phagosomal vesicle and then, utilize cytoskeletal actin polymerization for intracellular movement [86]. Numerous bacteria that are intracellular parasites of macrophages, like *Mycobacterium tuberculosis* and *Brucella abortus*, execute their escape through the destruction of macrophages [87,88]; however, their mechanisms are not fully understood. Based on the timeframe of escape, the mechanisms for those pathogens to circumvent phagocytosis may not be the same that *P. gingivalis* uses. A possible model for *P. gingivalis* W83 escape may involve the bacteria entering the macrophage in a putative phagosome and exiting this phagosome easier and at a higher rate than the other strains tested before the phagosome is fused with the lysosome. Takeuchi et al. (2011) have postulated that *P. gingivalis* can leave early endosomes and enter the recycling pathway (Rab11/RalA+ endosomes) to exit the host through exocytosis [80]. Accordingly, this model of entry/exit seems to coincide well with our 2-h timeframe of *P. gingivalis* W83 entry/exit from macrophages. Yet, this process will require further research to uncover the precise mechanisms of *P. gingivalis* escape from macrophages.

*P. gingivalis* is one of the major causative agents of periodontal disease and with this newly discovered ability to escape from macrophages, may have several clinical implications beyond the oral cavity. *P. gingivalis* is able to enter the bloodstream through sores and lesions within inflammation sites [89] and numerous studies have linked *P. gingivalis* to multiple systemic diseases [50,51,52,53,54,55,56,57,58,59,60,61,62,63,64,65,66,67,68]. The association between periodontitis and Chronic Obstructive Pulmonary Disease, an inflammation-driven respiratory disease with a high mortality rate, has been widely recognized in the past twenty years [62,64,90,91,92,93,94,95,96]. Another clinical manifestation linked to *P. gingivalis* systemic infection is insulin resistance and the development of diabetes [97,98,99,100,101,102,103]. Additionally, inflammation caused by chronic periodontitis and systemic *P. gingivalis* infection have been implicated in promoting the progression of Alzheimer’s disease, in susceptible individuals, through the activation of primed microglial cells by pro-inflammatory mediators [104,105,106,107,108,109,110,111,112]. Biomarkers malondialdehyde [69], asymmetric dimethylarginine, and c-reactive proteins may be used to monitor the development of both periodontitis and cardiovascular disease [69,70]. In addition, nutraceutical agents help ameliorate the clinical aspects of periodontitis [113], and could be related in the control of *P. gingivalis* escape. However, more research is necessary to understand this point. The ability of this pathogen to enter and exit macrophages within just a few hours can directly exacerbate all above clinical anomalies.

*P. gingivalis* has been shown to influence macrophage conversion to foam cells in the presence of high concentrations of low-density lipoproteins (LDL), typically in the bloodstream [68]. Foam cell formation occurs during macrophage uptake of modified LDL species like oxidized LDL [68]. LDL oxidation can occur due to oxidative stress from an imbalance of reactive oxygen species (ROS) production and antioxidant system activity, typically resultant from ROS overproduction induced by pathogens such as *P. gingivalis* [43,44,45,114,115]. The ability of *P. gingivalis* to enter, exit, and re-enter macrophages may result in larger amounts of foam cell propagation even with a small bacterial load, as one bacterium can infect and escape many macrophages (Figure 6, Table 2). In the *P. gingivalis* W83 strain, about 67.18% of the intracellular bacteria were able to escape M1 macrophages within 2 h. Of that, 6.42% were able to re-enter new macrophages (Figure 6, Table 2). The combination of high LDL levels and *P. gingivalis* in the bloodstream, as a result of periodontitis, may enhance foam cell propagation, increasing the risk of atherosclerosis. The accumulation of macrophage foam cells sets the stage for more complicated arterial plaques [34,43,44,50,51,52,53,55,57,59,68,114,115,116]. However, the cellular events that lead to macrophage foam cell formation upon a challenge with *P. gingivalis* warrant further investigation.

This study investigated *P. gingivalis* escape from cell-lines RAW 264.7 and THP-1-derived macrophages. Results using bone marrow-derived macrophages could be more indicative of host–bacteria interactions in the human body. Another enhancement to this study would be the testing of other *P. gingivalis* strains, including W50, 381, 23A4, A7A1, and HG169, as they are relevant to periodontitis [117]. Molecular similarities as well as differences between all these strains could help identify potential genes that may be involved in the escape process. It may also be beneficial to compare *P. gingivalis* to other oral pathogens like *Tannerella. forsythia*, *Aggregatibacter actinomycetemcomitans*, *Treponema denticola*, and *Fusobacterium nucleatum*, all of which are associated with periodontitis progression [118]. A comparison could help determine whether this entry, escape, and re-entry cycle is unique to *P. gingivalis* or if it is present in other oral pathogens. Another limitation is related to the types of techniques employed. In many studies, several techniques such as immunofluorescent staining and confocal microscopy have been used to visualize intracellular bacteria. Others use molecular probes and qPCR to quantify total bacterial loads. However, in this study, CFU counting was particularly selected as the main method to quantify intracellular and escaping bacteria because of several reasons: (i) CFUs account for live bacteria; (ii) the technique is extremely sensitive allowing the quantification of small numbers of bacteria; (iii) molecular or fluorescent probes would indicate the presence of bacteria, but not their viability; and (iv) microscopy would narrow the quantification of bacteria (live or dead) to the field of view. Although CFU counting is a less sophisticated technique, it serves the purpose of quantifying even a small number of live bacteria after escaping from macrophages.

Ultimately, our results indicate that *P. gingivalis* escapes from macrophages, the professional phagocytic cells of the immune system, at a significantly higher rate than other bacteria tested (Figure 5). Additionally, *P. gingivalis* exhibits a significant cycle of entry, exit, and re-entry in THP-1-derived M1 macrophages (Figure 6, Table 2). The deficiency of macrophages to capture and degrade *P. gingivalis* correlates with chronic in vivo infections in mice [80]. With the role of *P. gingivalis* in foam cell conversion, this escape and re-entry cycle implies that small infections can result in large amounts of foam cell propagation, where one bacterium can lead to multiple foam cells being formed. Further research will focus on identifying a possible mechanism of escape and perhaps shed light on pharmacological means to reduce escape ability and help mitigate chronic anomalies within the oral cavity as well as cardiovascular and systemic infections, including atherosclerosis.

## Figures and Tables

**Table 1:** Major genotypic and phenotypic differences between *P. gingivalis* W83 and 33277.

	W83	33277	References
**Major Fimbriae (genotype)**	Yes (type IV in genes only)	Yes (type I)	[75]
**Invasiveness**	High	Low	[75]
**Minor Fimbriae**	No	Yes	[75,76,77]
***PG0717***	Yes	No	[71]
**Gingipains (genotype)**	Kgp (I), RgpA (A), RgpB (NSSN, NYPN, NSSK—Possible association)	Kgp, RgpA no specific information on typing is available	[40,41]
**Classification**	Virulent	Avirulent	[78]
**Capsule**	Yes	No	[79]

**Table 2:** Entry, escape, and re-entry of bacteria in and out of THP-1-derived M1 cells.

Bacteria	Intracellular CFUs	Escaping CFUs ^a^	Total CFU Escape ^b^	Escaping and Re-Entering CFUs ^c^
***S. gordonii*** **DL1**	7030	125 (1.78%) 224 (3.18%)	349 (4.96%)	83 (23.78%)
***E. coli* OP50**	10,167	696 (6.85%) 412 (4.05%)	1108 (10.90%)	94 (8.48%)
***P. gingivalis* W83**	229,861	56,891 (24.75%) 97,530 (42.43%)	154,421 (67.18%)	9913 (6.42%)
***P. gingivalis* 33277**	23,722	2000 (8.43%) 1912 (8.06%)	3912 (16.49%)	1358 (34.71%)

All values in ( ) represent the percentage of CFUs from prior step as outlined in Figure 1. ^a^, top value = zero hours escape and bottom value = two hours of escape; ^b^, total escaping CFUs at 0 h and at 2 h; ^c^, total escaping bacteria from one set of M1 macrophages and re-entry into another set of M1 macrophages.
